# DCNet: DenseNet-77-based CornerNet model for the tomato plant leaf disease detection and classification

**DOI:** 10.3389/fpls.2022.957961

**Published:** 2022-09-08

**Authors:** Saleh Albahli, Marriam Nawaz

**Affiliations:** ^1^Department of Information Technology, College of Computer, Qassim University, Buraydah, Saudi Arabia; ^2^Department of Computer Science, University of Engineering and Technology–Taxila, Taxila, Pakistan; ^3^Department of Software Engineering, University of Engineering and Technology–Taxila, Taxila, Pakistan

**Keywords:** CornerNet, classification, DenseNet, tomato plant diseases, localization

## Abstract

Early recognition of tomato plant leaf diseases is mandatory to improve the food yield and save agriculturalists from costly spray procedures. The correct and timely identification of several tomato plant leaf diseases is a complicated task as the healthy and affected areas of plant leaves are highly similar. Moreover, the incidence of light variation, color, and brightness changes, and the occurrence of blurring and noise on the images further increase the complexity of the detection process. In this article, we have presented a robust approach for tackling the existing issues of tomato plant leaf disease detection and classification by using deep learning. We have proposed a novel approach, namely the DenseNet-77-based CornerNet model, for the localization and classification of the tomato plant leaf abnormalities. Specifically, we have used the DenseNet-77 as the backbone network of the CornerNet. This assists in the computing of the more nominative set of image features from the suspected samples that are later categorized into 10 classes by the one-stage detector of the CornerNet model. We have evaluated the proposed solution on a standard dataset, named PlantVillage, which is challenging in nature as it contains samples with immense brightness alterations, color variations, and leaf images with different dimensions and shapes. We have attained an average accuracy of 99.98% over the employed dataset. We have conducted several experiments to assure the effectiveness of our approach for the timely recognition of the tomato plant leaf diseases that can assist the agriculturalist to replace the manual systems.

## Introduction

In accordance with a report issued by the Food and Agriculture Organization (FAO) of the United Nations, the population of humans will undergo a tremendous increase around the globe to 9.1 billion by 2050. Such an increase in the number of humans will also raise the demand for food (Bruinsma, 2009). Meanwhile, the decrease in agricultural land and the unavailability of clean water will limit the progress of nutriment amounts. Therefore, there is an urgent demand for improving food yields by consuming minimum cultivation space to fulfill the necessities of humans. The occurrence of several crop abnormalities results in a substantial decline in both the yield and quality of food. Hence, the timely recognition of such plant diseases is required as these diseases can affect the profit of farmers and can increase the purchase cost of food. Such implications can introduce economic instability in the markets. Moreover, the plant diseases at their adverse stage can destroy the crops which can create a starvation scenario within a region, specifically in low-income countries. Plant inspections are generally carried out with the help of human experts. However, this is a cumbersome and time-consuming activity that relies upon the presence of area experts. These plant examination procedures are not considered very reliable and it is practically impossible for humans to inspect every plant separately (Pantazi et al., 2019). To enhance the quantity and quality of food, there is a need to timeously and correctly recognize the various plant diseases which can also force the farmers into using the costly spray methods. To tackle the above-mentioned problems of manual processes, the research community is focusing on the development of automated plant disease detection and classification systems (Wolfenson, 2013).

The focus of this paper is the recognition of several tomato plant diseases as tomato has the largest consumption rate, of 15 kg per capita within a year when compared to other vegetables such as rice, potato, and cucumber. Moreover, the tomato crop counts for 15% of the entire vegetable ingestion globally (Chowdhury et al., 2021). Further, tomatoes have the highest cultivation rate with an annual growth rate of 170 tons worldwide (Valenzuela and Restović, 2019). The leading countries for its production are Egypt, India, the United States, and Turkey (Elnaggar et al., 2018). In a study conducted by the FAO (Sardogan et al., 2018), the occurrence of several tomato plant diseases caused a severe reduction in its quantity and most of the abnormalities originated from the leaves of tomato plants. It has been observed that such diseases reduce the tomato food quantity from 8 to 10% annually (Sardogan et al., 2018). Farmers or agriculturalists can guard against these huge monetary losses by adopting automated systems which can assist them in the timely detection of plant diseases and taking proactive measures. At first, technology experts utilized the methods used in the field of molecular biology and immunology for locating the presence of tomato plant leaf diseases (Sankaran et al., 2010; Dinh et al., 2020). However, these techniques were not fruitful due to their high processing requirements and dependence on the expertise of humans. Most agriculturists belong to poor or under-developed countries where the adaptability of such an expensive solution is not affordable (Patil and Chandavale, 2015; Ferentinos, 2018). The rapid progression in the area of machine learning (ML) has introduced low-cost solutions for the recognition of tomato plant diseases (Gebbers and Adamchuk, 2010). Many researchers have tested the conventional ML methods, such as hand-coded approaches, in the field of agriculture (Gebbers and Adamchuk, 2010). The availability of economical image-capturing gadgets has assisted researchers to take pictures in real-time and then give intelligent predictions *via* using ML-based approaches. Examples of such approaches include K-nearest neighbors (KNN), decision trees (DT) (Rokach and Maimon, 2005), and support vector machines (SVM) (Joachims, 1998), which are heavily evaluated by researchers for plant disease classification. Such techniques are simple in their architecture and can work well with a small amount of training data. However, they are unable to contend with image distortions such as intensity variations, color changes, and brightness alterations of suspected samples. Furthermore, the conventional approaches always impose a trade-off among the classification performance and processing time (Bello-Cerezo et al., 2019).

The empowerment of DL frameworks has assisted the researchers in dealing with the problems of conventional ML approaches (Agarwal et al., 2021d,^2022^). Several DL techniques such as CNN (Roska and Chua, 1993), recurrent neural networks (RNNs) (Zaremba et al., 2014), and long short-term memory (LSTM) (Salakhutdinov and Hinton, 2009) have been found to be reliable in recognizing plant leaf diseases. The DL approaches are inspired by the human brain and can learn to discriminate between a set of image features without relying on the intervention of domain experts. These frameworks recognize the objects in the same way as humans by visually examining several samples to accomplish a pattern recognition task. Because of such properties, the DL approaches are found to be more suitable in areas of agriculture, including plant disease classification (Gewali et al., 2018). Several well-known DL frameworks such as GoogLeNet (Szegedy et al., 2015), AlexNet (Yuan and Zhang, 2016), VGG (Vedaldi and Zisserman, 2016), and ResNet (Thenmozhi and Reddy, 2019) have been thoroughly tested for accomplishing several jobs in farming, i.e., estimating food yield, crop heads recognition, fruit totaling, plant leaf disease detection and categorization, among others. Such approaches show reliable performance by minimizing the processing complexity as well as by better analyzing the topological information of the input samples.

Numerous techniques have been evaluated to identify and classify tomato leaf diseases. However, the reliable and timely recognition of such abnormality is a complicated job because of the significant color resemblance between the healthy and diseased areas of plant leaves (Paul et al., 2020). Furthermore, the intense changes in the dimension of plant leaves, lightning conditions, the incidence of noise, and blurring in the input samples further problematize the disease recognition procedure. Hence, there is a need for a more reliable system to accurately perform the plant disease classification process with minimum time constraints. To deal with these issues, we have introduced a DL approach, namely the custom CornerNet model. We have utilized Dense-77 as the backbone of the CornerNet model for extracting the image features. These are later classified by the one-stage detection module of the CornerNet model. We have conducted extensive evaluation over a challenging dataset and confirm that our approach is proficient in classifying the numerous types of tomato plant leaf diseases. The major contributions of the proposed approach are listed as:

1. Modified an object detection approach named CornerNet for tomato plant leaf abnormality categorization which improves the classification performance with an accuracy value of 99.98%.
2. Exhibits robust performance for 10 classes of the tomato plant leaf diseases because of the empowerment of the custom CornerNet model to tackle the over-fitted model training data.
3. A cost-effective solution is presented for the classification of tomato plant leaf abnormalities which minimizes the test time to 0.22 s.
4. Efficient localization of diseased regions from the tomato plant samples due to the better keypoints calculation power of the Dense-77-based CornerNet model with the mean average precision (mAP) value of 0.984.
5. In contrast to several new methods, extensive experimentation has been carried out on a challenging database named the PlantVillage dataset to exhibit the robustness of the proposed work.
6. The presented work is capable of correctly identifying the abnormal area of the tomato plant leaves even from the distorted samples and under the influence of size, color, and light variations.

The article is structured as follows: existing studies are compared in section “Related work,” the details of the introduced approach are described in section “Materials and methods,” section “Results” contains the results, and the conclusion is drawn in section “Conclusion.”

## Related work

In this section, we review existing studies that have attempted to classify tomato plant leaf diseases. Typically, the approaches for tomato plant leaf disease detection and classification are either conventional ML-based techniques or DL frameworks. Hand-coded features computation approaches with the ML-based classifiers were explored initially for the plant leaf disease classification. One such framework was presented in Le et al. (2020) where the suspected images were initially processed by applying the morphological opening and closing techniques to remove the undesired objects. Then, the filtered local binary pattern method, namely the k-FLBPCM, was used on the processed images to obtain the desired feature vector. Finally, the SVM classifier was trained on the computed features for classification. The technique in Le et al. (2020) improved classification results for the plant leaf diseases but was unable to show better results on the distorted samples. Another work, namely Directional Local Quinary Patterns (DLQP), was introduced in Ahmad et al. (2020) to extract the keypoints from the input images. The work also used the SVM classifier on the computed features for categorizing the several classes of plant leaf diseases. The solution introduced in Ahmad et al. (2020) was robust in classifying the affected areas of plant leaves into their respective groups but classification performance degraded for noisy images. Sun et al. (2019) proposed an automated solution to quickly locate the diseased portion of plant leaves. They used the Simple Linear Iterative Cluster (SLIC) algorithm for distributing the input images into numerous chunks. Then, for each block of the divided image, the GLCM approach was used to extract the features which were later combined and passed to the SVM classifier for classification. This approach (Sun et al., 2019) performed well in recognizing the several categories of plant diseases but suffered from extensive processing complexity. Another pattern recognition approach was used in Pantazi et al. (2019) where the input sample was initially segmented *via* applying the GrabCut method to locate the region of interest. Then, the LBP algorithm was applied for keypoints vector estimation. Finally, classification was carried out with the help of the SVM classifier. This technique (Pantazi et al., 2019) was proficient in locating the abnormal area of the plant leaves. However, detection performance degraded for the samples with intense noise attacks. Ramesh et al. (2018) proposed a computer-aided system for the automated detection and classification of several abnormalities of plant leaves. For feature estimation, the HOG filter was used on the input samples, and disease classification was performed using the Random Forest (RF) technique. This work, elaborated on in Ramesh et al. (2018), was found to be a lightweight solution for the recognition of plant leaf diseases but the classification accuracy required further improvements. Another technique was discussed in Kuricheti and Supriya (2019) where an ML-based approach was presented to classify the several abnormalities of the turmeric plant. In the first phase, the K-means clustering approach was used on the input sample to locate the area of interest. The GLCM algorithm was applied to this area to calculate the feature vector. Finally, the SVM classifier was adopted for classification using the computed keypoints. The work discussed in Kuricheti and Supriya (2019) showed better plant leaf disease classification results. However, detection performance degraded for images with large brightness changes. Another handcrafted feature estimation approach to recognize and categorize crop leaf diseases was found in Kaur and Education (2021). Several pattern-based approaches like the GLCM, LBP, and SIFT were used for feature vector estimation. Then, several well-known ML classifiers, named the SVM, RF, and KNN, were trained on the computed features to execute the classification task. The best results were reported for the RF classifier but the classification accuracy needed enhancement. A similar solution was elaborated on in Shrivastava and Pradhan (2021) where the fourteen color spaces approach was used to extract the keypoints from the test images with a length of 172. Then, the calculated keypoints were passed to the SVM algorithm to classify the samples into their respective classes based on the detected abnormal plant leaf areas. This solution (Shrivastava and Pradhan, 2021) provided superior plant leaf disease categorization results. However, this performance degraded for samples with significant color and light changes.

Due to the empowerment of DL frameworks and their ability to better deal with image transformations, researchers are now employing them for recognizing plant diseases.

The framework in Argüeso et al. (2020) used the DL technique named Few-Shot Learning (FSL) for recognizing the affected portions of crops and determining the related category. The InceptionV3 model was applied to capture the keypoints of the input image. The SVM classifier was used to classify the samples using the keypoints, according to the detected disease. The approach described in Argüeso et al. (2020) exhibited robust plant disease classification results but requires extensive data for the model training. Agarwal et al. (2020b) proposed a CNN framework containing 3 convolution layers as the feature extractor module before classification. The framework presented in Agarwal et al. (2020b) was a lightweight solution for the plant leaf disease classification but performance degraded for noisy samples. Another lightweight model was presented in Richey et al. (2020) to be used with cellphones. The ResNet50 approach was used as the end-to-end framework to compute the deep features and perform the classification task. The approach improved the processing complexity for plant disease classification. However, it was not supported by all mobile phones due to the memory requirements. Another framework was depicted in Batool et al. (2020) to classify the numerous types of tomato crop abnormalities. The AlexNet model was employed to extract the deep features of the plant images which were later passed as input to the KNN approach for the classification of the images into their respective category. This work was proficient in recognizing the various categories of tomato plant leaves. However, the KNN algorithm was a time-consuming approach. Similarly, an approach for categorizing the tomato plant leaf abnormalities was described in Karthik et al. (2020) that employed the residual method to compute the reliable feature set. A CNN-based classifier was introduced to categorize the samples based on the learned features of different classes. The approach (Karthik et al., 2020) classified the samples in the related categories better. However, it required a large number of samples for training, which further complicated the model. Dwivedi et al. (2021) applied the object detection approach named region-based CNN (RCNN) to automatically detect and localize the diseased area of grape plant leaves. The approach used the ResNet18 as the feature extractor unit which calculates the keypoints set from the plant images. In the next phase, the RCNN framework applied the region proposals approach to locate the affected portion and determine the associated class. The solution depicted in Dwivedi et al. (2021) worked well in recognizing the various diseases of the grape plant but was unable to generalize well from unseen training data. Another approach was discussed in Akshai and Anitha (2021) where several DL frameworks, namely VGG, DenseNet, and ResNet, were evaluated for the detection and classification of several types of plant leaf diseases. This approach (Akshai and Anitha, 2021) showed better results for the DenseNet model. Albattah et al. (2021) proposed an object detection approach, namely the CenterNet model, for the automated identification and classification of numerous types of plant leaf diseases. Initially, the dense model was used for the extraction of the keypoints set from the input images. These were then used to recognize the diseased portion of plant samples. This approach (Albattah et al., 2021) showed better plant leaf abnormality recognition ability. However, the model needed assessment on a more challenging dataset. Another DL approach was evaluated in Albattah et al. (2022) where the EfficientNetV2 model was tested for the classification of numerous types of plant diseases, that results in improving the classification performance. In Agarwal et al. (2021c), a DL approach, namely the VGG16 model, was used in the classification of tomato leaf diseases. The approach introduced the concept of model optimization, but the detection performance required extensive result improvements. Similarly, other works discussed the model optimization concept for the plant leaf diseases categorization (Agarwal et al., 2021a,b) but the recognition results needed improvement. Zhao et al. (2021) presented a model to recognize numerous tomato plant leaf abnormalities in which the CNN approach, merged with an attention mechanism, was utilized. The methodology attained classification results of 99.24%. Moreover, in Maeda-Gutiérrez et al. (2020), different DL networks, i.e., Inception V3, AlexNet, GoogleNet, ResNet-18, and SE-ResNet50 were tested for tomato plant disease classification. The GoogleNet approach worked well with classification results of 99.39%. Bhujel et al. (2022) also proposed a DL model, namely ResNet18, along with the CBAM for recognizing the tomato plant abnormalities and achieved an accuracy of 99.69%. The methods in Maeda-Gutiérrez et al. (2020), Zhao et al. (2021), and Bhujel et al. (2022) enhanced the tomato plant leaf diseases categorization results. However, these works accomplished classification at the image level and are incapable of identifying the precise diseased area.

A critical investigation of existing work is outlined in Table 1, which depicts that there is a performance gap that requires a more reliable model. This model must be proficient enough to recognize the numerous categories of tomato plant leaf disease and minimize the time complexity. In the presented work, we have tried to cover this gap by proposing a more accurate and robust approach for tomato plant leaf disease classification.

**TABLE 1:** An analysis of existing methods.

Reference	Method	Accuracy (%)	Limitation
**Hand-coded approaches**			
Le et al., 2020	K-FLBPCM + SVM	98.63	The technique lacks the ability to classify distorted plant images.
Ahmad et al., 2020	DLQP + SVM	97.80	This approach is not efficient for noisy images.
Sun et al., 2019	GLCM + SVM	98.50	The technique entails high computational costs.
Pantazi et al., 2019	LBP + SVM	95	This approach is not efficient for noisy images.
Ramesh et al., 2018	HOGs + RF	70.14	The work requires classification result improvements.
Kuricheti and Supriya, 2019	GLCM + SVM	91	The technique lacks the ability to tackle the intensity and color variations found in the plant images.
Kaur and Education, 2021	SIFT, LBP, GLCM + SVM, KNN, and RF	82.12	The results need further improvements.
Shrivastava and Pradhan, 2021	Color spaces + SVM	94.65.	The approach is not robust for unseen data.
**DL approaches**			
Argüeso et al., 2020	InceptionV3 + SVM	91.40	The technique needs further assessment over a more complex database.
Agarwal et al., 2020b	CNN	91.20	The framework is facing the network over-fitting problem.
Richey et al., 2020	ResNet50	99	The approach requires high processing power.
Batool et al., 2020	AlexNet + KNN	76.10	The approach takes a long time to process samples.
Karthik et al., 2020	CNN	98	The work needs huge samples to train the network.
Dwivedi et al., 2021	RCNN	99.93	The approach does not perform well for unseen examples.
Akshai and Anitha, 2021	VGG, ResNet, and DenseNet	98.27	The approach requires high processing power.
Albattah et al., 2021	CenterNet	99.90	The framework needs to be evaluated on real-world examples.
Albattah et al., 2022	EfficientNetV2	99.93	Performance degrades for distorted samples.
Agarwal et al., 2021c	VGG16	98.40	The classification accuracy requires improvements.

## Materials and methods

**FIGURE 1:**
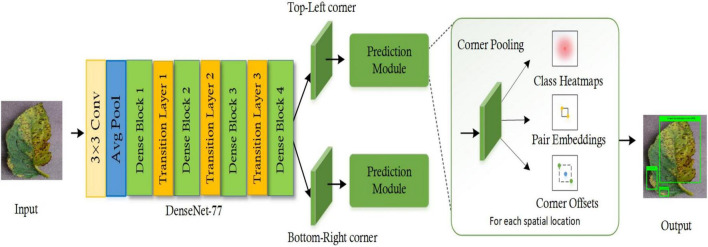
Pictorial depiction of the DenseNet-77-based CornerNet model for the tomato plant leaf diseases classification.

In this section, an in-depth discussion of the proposed technique for tomato plant leaf disease localization and classification is presented. The basic motivation of this framework is to present an accurate and computationally efficient approach that is empowered to automatically nominate a representative feature vector independent from executing any manual examination. Our work comprises two main steps to accomplish the automated recognition of plant leaf diseases. First, the images from the PlantVillage dataset are employed to develop the annotations to correctly identify the affected portions and their associated classes. Then, these annotations are used in training the DenseNet-77-based CornerNet approach. During the test phase, the images from the test set are used to validate the model’s performance. More precisely, we have customized the CornerNet model (Law and Deng, 2019) by introducing the DenseNet-77 network in its feature extraction unit. The DenseNet-77 approach as the base network computes the feature vector which is then passed to the one-stage detector of the CornerNet model to localize and classify the affected regions into 10 classes. Several standard evaluation measures are then used to quantitatively measure the performance of the introduced framework. The detailed model formulation of our framework is given in Algorithm 1, while the pictorial demonstrations showing the detailed steps of our approach are given in Figure 1.

**Algorithm 1:** Description of steps followed by the proposed work.

**INPUT:** TS, AI **OUTPUT:** *Bbx*, CustomCoNet, C-score ***TS*** - total no of samples used for model training **AI** - annotated images showing the diseased area on the tomato plant leaves ***Bbx*** - rectangular box showing the diseased region on the output image ***CustomCoNet*** - CornerNet model with the DenseNet-77 backbone **C-score** - confidence score along with predicted class **SampleSize ← [x y]** **Bbx computation** β ← **AnchorsCalculation (*TS*, AI)** **CustomCustomCoNet-Model** **CustomCoNet** ← CornerNetWithDenseNet-77 (*SampleSize*, β) **[*d***_r_ ***d***_t_] ← Distribution of dataset into *train and test sets* **The training module for tomato plant leaf disease detection and classfication** **Foreach** image **m** in → **d**_r_ **Calculate** *DenseNet-77-based-*deepFeatures ← *df* **End For** Train **CustomCoNet** on *df*, and measure network training time as ***t_d77*** β _***dense*** ← EstimateDiseasedPos(df) ***V**_**dense*** ← Validate_Model (*DenseNet*-77, β_*dense*) **Foreach** images ***M*** in → **d**_t_ (**i**) Measure features with trained model €→***V**_**dense*** (**ii**) [*Bbx, C-score, class*] ← Predict (€) (**iii**) Present output image with B*box, class* (**iv**) η ← [η bbox] **End For** ***Ap***_**€** ← Test framework € using η ***Output_class*** ← CustomCoNet (*Ap*_€).

### Data preparation for model training

**FIGURE 2:**
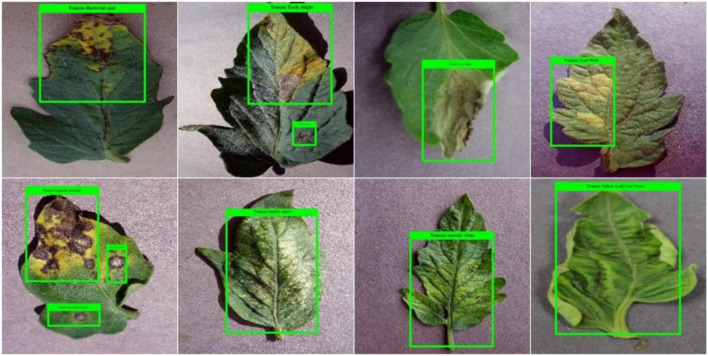
Example of annotated images of the tomato plant from the PlantVillage dataset.

The training of the object detection model was based on annotations development. This was focused on clearly localizing the affected region from the training samples and their associated category. Therefore, in the first step, we have used the images from the training set of the plant samples from the PlantVillage dataset and used the LabelImg software (Lin, 2020) for relevant annotation generation. These annotations assist in exactly outlining the diseased areas of leaves by developing the bounding box (*bbx*) around them. The dimensions of the annotations are saved as an XML file which is later employed for model training. A few examples of annotated samples are given in Figure 2.

### CornerNet model

The CornerNet (Law and Deng, 2019) is a well-known one-stage object detection model that recognizes the region of interest (ROI) (the diseased region of the tomato plants in this case) from the input samples through keypoint calculation. The CornerNet model estimates the Top-Left (T_L_) and Bottom-Right (B_R_) corners to draw the *bbx* with more accuracy when compared to other object detection models (Girshick, 2015; Ren et al., 2016). The CornerNet framework is comprised of two basic units: the feature computation backbone and the prediction module (Figure 1). At the start, a keypoints extractor unit is used which extracts the reliable feature vector that is employed to estimate the heatmaps (*Hms*), embeddings, offset, and class (*C*). The *Hms* give an approximation of a location in a sample where a T_L_/B_R_ corner is associated with a particular category (Nawaz et al., 2021). The embeddings are used to discriminate the detected pairs of corners and offsets to fine-tune the *bbx* position. The corners with high scored T_L_ and B_R_ coordinates are used to determine the exact position of the *bbx*, whereas the associated category for each detected diseased region is specified by using the embedding distances on the computed feature vector.

The CornerNet framework shows robust performance in detecting and classifying several types of objects (Girshick, 2015; Raj et al., 2015; Redmon et al., 2016; Zhao et al., 2016). However, the abnormalities of tomato plant leaves have some distinct characteristics. These include leaves of different shapes and sizes and high color resemblance in the affected and healthy regions of plant leaves which complicates the classification procedure. Moreover, the existence of several image distortions such as differences in the light, color, and brightness of the samples and the incidence of noise and blurring effect further increase the complexity of the tomato plant leaf disease classification process. Therefore, to better tackle the complexities of samples, we have customized the CornerNet model by introducing a more effective feature extractor, namely the DenseNet-77, as its base network. The introduced base network is capable of locating and extracting the more relevant sample attributes which assist the CornerNet approach and enhance its recall ability in comparison to the conventional model.

The reason for selecting the CornerNet approach for classifying the diseases of tomato plants in this study is its capability for effectively detecting objects by utilizing keypoint approximation in comparison to earlier approaches (Girshick, 2015; Girshick et al., 2015; Liu et al., 2016; Ren et al., 2016; Redmon and Farhadi, 2018). The framework utilizes detailed keypoints and identifies the object by employing a one-stage detector. This eliminates the need to use large anchor boxes for diverse target dimensions as used in other one-stage object recognition models, i.e., single-shot detector (SSD) (Liu et al., 2016), and You Only Look Once (YOLO) (v2, v3) (Redmon and Farhadi, 2018). Moreover, the CornerNet model is more computationally robust than the other anchor-based two-stage approaches, i.e., RCNN (Girshick et al., 2015), Fast-RCNN (Girshick, 2015; Nazir et al., 2020), and Faster-RCNN (Ren et al., 2016; Albahli et al., 2021), as these techniques employ two phases to accomplish the object localization and categorization. Consequently, the DenseNet-77-based CornerNet framework efficiently deals with the issues of existing models by presenting a more proficient network that extracts more nominative sample features and reduces the computational cost.

### Modified CornerNet framework

The base of a model is responsible for identifying and computing the reliable feature vector that gives the semantic information and reliable location of a target in an image. The affected regions of tomato plant leaves are small, therefore a robust and representative set of keypoints is mandatory to recognize the diseased portion from complex backgrounds such as changing acquisition positions, lightning conditions, noise, and blurring. The conventional CornerNet approach was introduced along with the Hourglass104 as the base network (Law and Deng, 2019). The major drawback of the Hourglass104 network is its huge structural complexity. The larger number of framework parameters increases the computational burden on the CornerNet model and slows down the target identification procedure. Further, the Hourglass104 approach is inefficient when computing reliable keypoints for all types of image distortions, e.g., extensive changes in the size, color, and orientation of the affected areas (Zhao et al., 2019). Therefore, we have changed the feature extractor layer of the CornerNet model to enhance the identification and categorization performance for tomato plant leaf diseases. To this end, we have utilized the DenseNet-77 (Huang et al., 2017) as the base network of the CornerNet model in our proposed approach.

#### DenseNet-100 feature extractor

**FIGURE 3:**
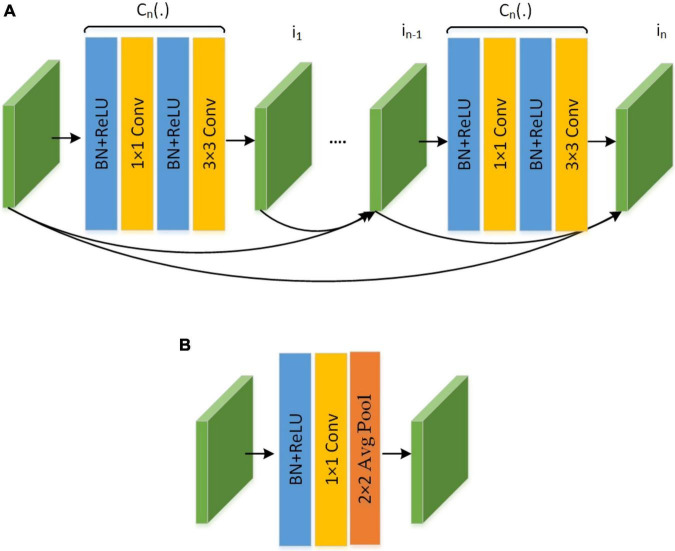
The pictorial representation of **(A)** dense block and **(B)** transition block.

The DenseNet-77 network is a lightweight model from the DenseNet family and has two major benefits over the conventional DenseNet approach: first, the number of model parameters is smaller than the original DenseNet model (Masood et al., 2021); secondly, the layers within each dense block (D_b_) are also reduced to further simplify its structure. The employed DenseNet-77 model is a shallower framework compared to the Hourglass104 approach and comprises four D_*b*_s in total. A detailed demonstration of the architectural representation of the DenseNet-77 is given in Figure 3. The DenseNet-77 approach comprises a smaller number of model parameters (6.2M) in comparison to the Hourglass104 base network (187M). Such architectural settings give it a computational advantage over the original base network. In all D_b_s, the convolution layers are directly linked and the computed feature maps from starting layers are communicated to the subsequent layers. The DenseNet model encourages the reemployment of the computed features and facilitates the communication of the computed data in the entire network structure. This empowers it to deal with the image distortions effectively (Huang et al., 2017). Table 2 shows the network depiction of the DenseNet-77 model.

**TABLE 2:** Description of the DenseNet-77.

Layer	DenseNet-77	
	Size	Stride
CnL 1	7×7 *cn*	2
Pool_L_1	3×3_max_pooling_	2
Db 1	$\left[\begin{array}{cc} 1\times 1 & cn\\ 3\times 3 & cn \end{array}\right]\times 6$	1
TnL		
Cn_L_2	1×1 *cn*	1
Pool_L_2	2×2*Ap*_L_	2
Db 2	$\left[\begin{array}{cc} 1\times 1 & cn\\ 3\times 3 & cn \end{array}\right]\times 12$	1
TnL		
Cn_L_3	1×1 *cn*	1
Pool_L_3	2×2_ApL_	2
D_b_3	$\left[\begin{array}{cc} 1\times 1 & cn\\ 3\times 3 & cn \end{array}\right]\times 12$	1
Tn_L_		
Cn_L_4	1×1 *cn*	1
Pool_L_4	2×2*Ap*_L_	2
D_b_4	$\left[\begin{array}{cc} 1\times 1 & cn\\ 3\times 3 & cn \end{array}\right]\times 6$	1
Classification_layer	7×7*Ap*_L_	
	FCL	
	SoftMax	

The network consists of numerous Convolutional Layers (Cn_L_), D_b_s, and Transition Layers (Tn_L_). A pictorial depiction of the D_b_ is given in Figure 3 and is the fundamental part of the DenseNet framework. In Figure 3, *i*_0_ represents the input layer and *k*_0_ depicts the feature maps. Furthermore, *C*_*n*_(.) is a compound function containing 3 consecutive actions: a 3 × 3 Cn_L_ filter, Batch Normalization (Bt_N_), and ReLU. Each *C*_N_(.) operation produces keypoint maps (*k*), that are used as input *i*_N_ succeeding layers. The employment of all earlier computed features to the next layers introduces the *k* × (*t−*1)+*k*_0_ feature maps at the *t*-th layer of D_b_, which increases the feature space immensely. Hence, the Tn_L_ is used between the D_b_ to lessen the computed features. The Tn_L_ is calculated as Bt_N_ and 1 × 1 Cn_L_ and the average pooling layer is represented as Ap_L_, as depicted in Figure 3.

#### Prediction module

The feature computation framework consists of two separate output units that denote the T_L_ and the B_R_ corners estimation branches, respectively. Each branch unit comprises a corner pooling layer (CP_L_) positioned on the top of the backbone to pool keypoints and produces three results: *Hms*, embeddings, and offsets. The prediction module is an improved residual block (RB) containing two 3 × 3 Cn_L_ and one 1 × 1 residual network, followed by a CP_L_. The CP_L_ assists the framework to identify the potential corners. The reduced keypoints are used as the input into a 3 × 3 Cn_L_-Bt_N_ layer and then the reverse projection is performed. This improved RB is followed by a 3 × 3 Cn_L_ which produces *Hms*, embeddings, and offsets. The *Hms* give the approximation of a location in a sample, as a T_L_/B_R_ corner, that is associated with a particular category. The embeddings are used to discriminate between the detected pairs of corners and offsets to fine-tune the *bbx* position. A suspected image can contain more than one affected region, therefore, embeddings assist the model to determine if the predicted corner points belong to a single disease class or different disease classes.

#### Detection

The CornerNet model is a deep learning framework that is independent of the selective search and proposal generation techniques. The test image and the associated annotated sample are given as input to the trained model. The improved CornerNet model extracts the corner points for the diseased area of the tomato plants and computes the associated offsets to the *x* and *y* coordinates, the measurements of *bbx*, and the associated class.

#### Loss function

The employed framework for the detection and classification of tomato leaf disease is an end-to-end learning method that practices multi-task loss during the training to increase its recognition ability and precisely locate affected leaf regions. The total training loss, designated by *L*_t_, is the combination of four different losses, given as:


(1) $$L_{t}=L_{d}+\alpha L_{pl}+\beta L_{ps}+\gamma L_{off}$$

Here, the *L*_d_ signifies detection loss accountable for corner identification, while *L*_*pl*_ denotes the group loss of group corners of the same *bbx*. Moreover, *L*_*ps*_ is the corner separation loss used to separate the corners of different *bbx*, and *L*_*off*_ is the smooth *L*1 loss designated for offset adjustment. The symbols α, β, and γ are the constants for our approach, with the values of 0.1, 0.1, and 1, respectively. The mathematical description of the *L*_*d*_ is given in Eq. 2.


(2) $$L_{d}=\frac{-1}{R}\sum_{j=1}^{\text{c}}\sum_{u=1}^{h}\sum_{v=1}^{\text{w}}\left\{ \begin{array}{c} {\left(1-t\right)}^{\emptyset }\log\left(t\right)\;if\left(g\right)=1\\ {\left(1-g\right)}^{\omega }t^{\emptyset }\log\left(1-t\right)\;otherwise \end{array}\right.$$

In this equation, *R* is the total number of detected diseased areas in a given image. For a given image, *c*, *h*, and *w* designate its total channels, width, and height. Moreover, *t*_*juv*_ indicates the estimated score at a given position (*u, v*) for the diseased area of class (*j*) in the suspected sample, and *g*_juv_ is the related ground-truth value. The ∅ and ω indicates the model hyperparameters that govern the influence of every selected point and have the values of 2 and 4 for our framework, respectively.

In downsampling, the dimension of the output sample is reduced than the actual sample size. The position (*u, v*) of the diseased portion in the test sample is plotted to the position $\left(\frac{u}{N},\frac{v}{N}\right)$ in the *Hms*, where *N* indicates the downsampling factor. The remapping of *Hms* to the actual sample size introduces precision loss that eventually degrades the IoU performance for small *bbx.* To tackle this problem, the offsets for all locations are computed to fine-tune the corner dimensions as described in Eq. 3.


(3) $$O_{i}=\left(\frac{u^{i}}{N}-\left\lfloor \frac{u^{i}}{N}\right\rfloor ,\frac{v^{i}}{N}-\left\lfloor \frac{v^{i}}{N}\right\rfloor \right)$$

Here, *O*_i_ shows calculated offset, while for corner *i*, *u^i^*, and *v^i^* represents the coordinators of *u* and v. Furthermore, the *L*_*off*_, employs the smooth *L1* method for adjusting the corner positions and is represented as:


(4) $$L_{off}=\frac{1}{M}\sum_{i=1}^{M}SmoothL1Loss\left(O_{i},0_{i}^{\prime }\right)$$

There could be several affected regions on a single image. Therefore, several B_R_ and T_L_ corners are nominated. For all corners, the model estimates an embedding vector to decide whether a group of B_R_ and T_L_ corners is associated with the same disease class or different disease classes. For this purpose, the CornerNet model uses the “pull and push” losses for framework training and are given as:


(5) $$L_{pl}=\frac{1}{M}\sum_{x=1}^{M}\left[{\left(e_{lx}-e_{x}\right)}^{2}+{\left(e_{rx}-e_{x}\right)}^{2}\right]$$

(6) $$L_{ps}=\frac{1}{M\left(M-1\right)}\sum_{x=1}^{M}\sum_{y=1,y\neq x}^{M}\max\left[0,\Delta -|e_{x}-e_{y}\right|$$

Here, *e*_lx_ shows the T_L_ while the *_e_rx_* denotes the B_R_ corners for a diseased region *x* and *e*_x_ is the average value of *e*_rx_ and *e*_rx_. The distance value to declare two detected corners belonging to different categories is set as 1, while the value of Δ is also 1 for all experiments.

## Results

In this section, we will outline detailed information about the dataset employed for the detection and classification of tomato plant leaf diseases. Moreover, the mathematical description of the used performance measures is also given. Finally, the results of the extensive experiments that have been conducted to show the efficacy of the proposed approach for tomato plant leaf disease recognition will be discussed.

### Dataset

**FIGURE 4:**
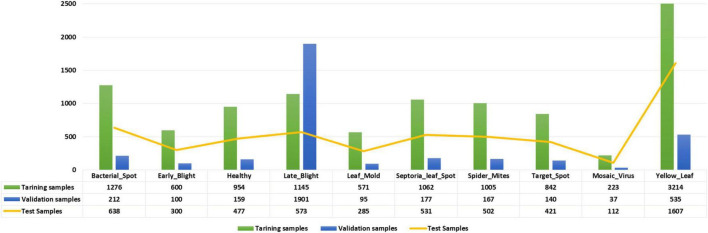
Details of the tomato plant samples from the PlantVillage dataset.

**FIGURE 5:**
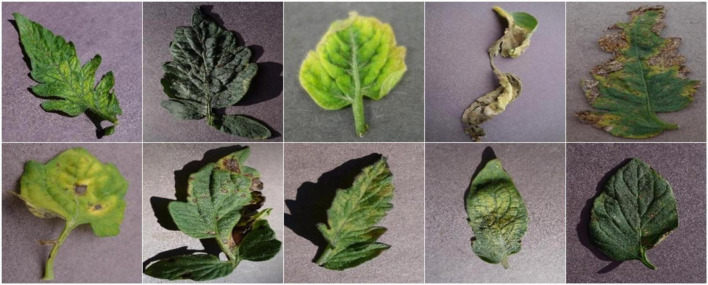
An example of tomato plant leaves samples from the PlantVillage dataset.

We have used the PlantVillage database (Hughes and Salathé, 2015), a large repository accessible online, to evaluate the effectiveness of the model in detecting and classifying tomato leaf diseases. This dataset is comprised of a total of 54,306 images for 14 crop types. As this study is focused on the diseases of the tomato plant, we have utilized the tomato plant samples belonging to 10 different diseases. The main reason to employ the PlantVillage dataset for our work is that its images contain severe alterations in the size, chrominance, and position of the affected leaf regions. Furthermore, the images contain noise, brightness changes, blurring, and color alterations. An in-depth demonstration of the employed dataset is elaborated in Figure 4 while a few samples are shown in Figure 5.

### Performance measures

**FIGURE 6:**
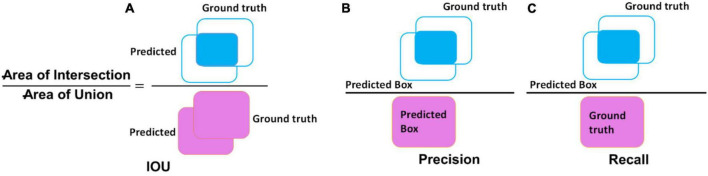
Visual demonstration of **(A)** IOU, **(B)** precision, and **(C)** recall.


For measuring the performance of the custom CornerNet model in detecting and classifying tomato plant leaf diseases, we have selected several standard metrics such as accuracy, mAP, intersection over union (IOU), precision, and recall. The mathematical description of accuracy and the mAP measure are given in Eqs 7, 8, respectively, while a graphical demonstration of precision, recall, and IOU is given in Figure 6.

(7) $$Accuracy=\frac{TP+TN}{TP+FP+TN+FN}$$

(8) $$mAP:=\sum_{i=1}^{T}AP\left(t_{i}\right)/T$$

### Localization results

**FIGURE 7:**
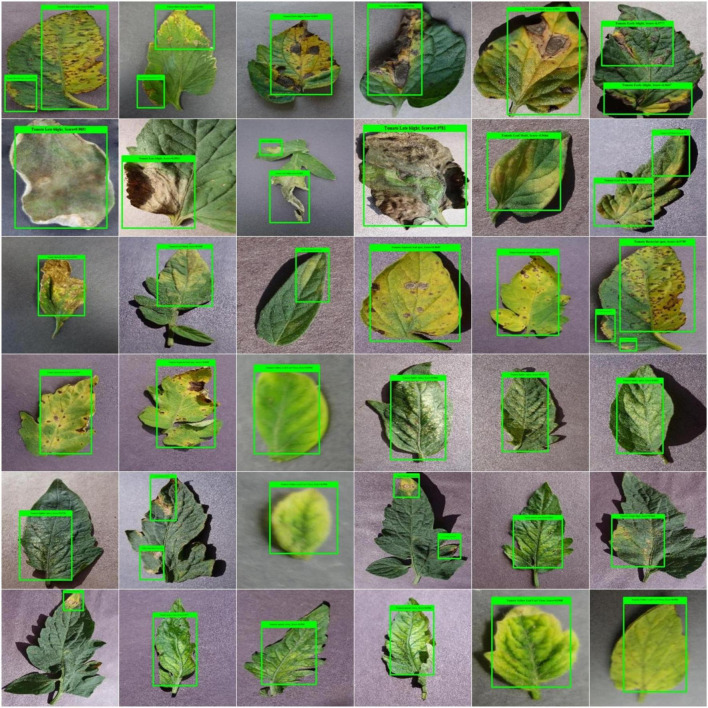
A pictorial depiction of the localized tomato plant leaf diseases samples.

The distinguishing attribute of a robust plant leaf disease classification framework is its ability to differentiate among the different classes of disease. To measure this, we designed an experiment. To visually elaborate on the detection performance of the custom CornerNet model, we have depicted the localized samples from the used dataset in Figure 7. The samples in Figure 7 clearly show that our technique is quite efficient in detecting the affected portion of the plant leaves and recognizing the associated classes even under the incidence of color, size, light, chrominance, and brightness changes.

The high recall power of the custom CornerNet model allows it to appropriately identify and categorize the several classes of tomato plant abnormalities. To numerically show the robustness of the proposed solution for tomato plant leaf disease classification, we have used two measures, namely the mAP and IOU score. These are the standard and most heavily employed metrics by the research community for object detection models. The proposed CornerNet model has localized the diseased portion from the plant samples with mAP and IOU scores of 0.984, and 0.979, respectively, which shows the effectiveness of our approach.

### Classification performance

**FIGURE 8:**
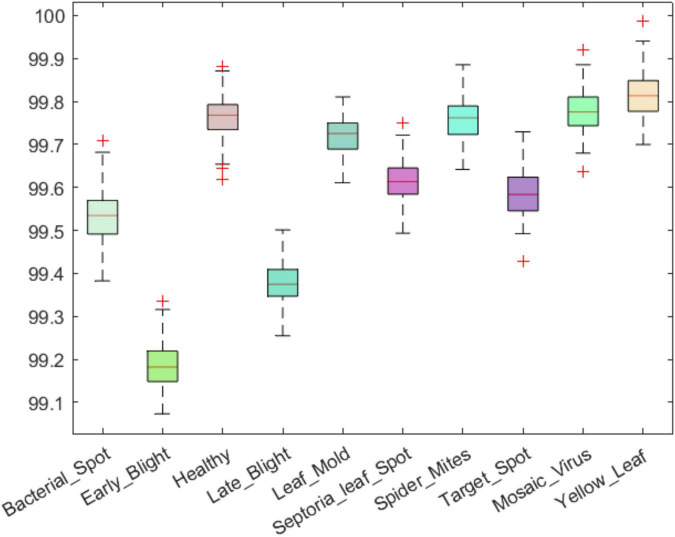
A pictorial depiction of the class-wise precision values obtained for the DenseNet-77-based CornerNet model.

**FIGURE 9:**
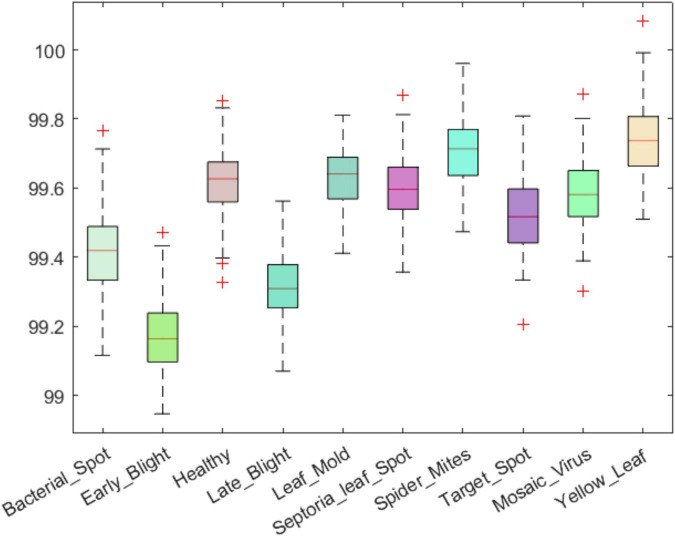
A pictorial depiction of the class-wise recall values obtained for the DenseNet-77-based CornerNet model.

An efficient plant leaf disease recognition system must be powerful enough to accurately discriminate among the different types of diseases. We tested the class-wise performance of the presented model with the help of several standard metrics such as precision, recall, accuracy, and F1-score. Initially, we computed the precision and recall values for the custom CornerNet model in locating and classifying the 10 categories of plant leaf abnormalities. We have used the boxplot to show the obtained results as these plots provide a better understanding of the results by showing the minimum, maximum, and average values for the employed metrics (Figures 8, 9). The results reported in Figures 8, 9 show that the introduced approach is capable of correctly classifying the 10 classes of tomato plant leaf diseases.

**FIGURE 10:**
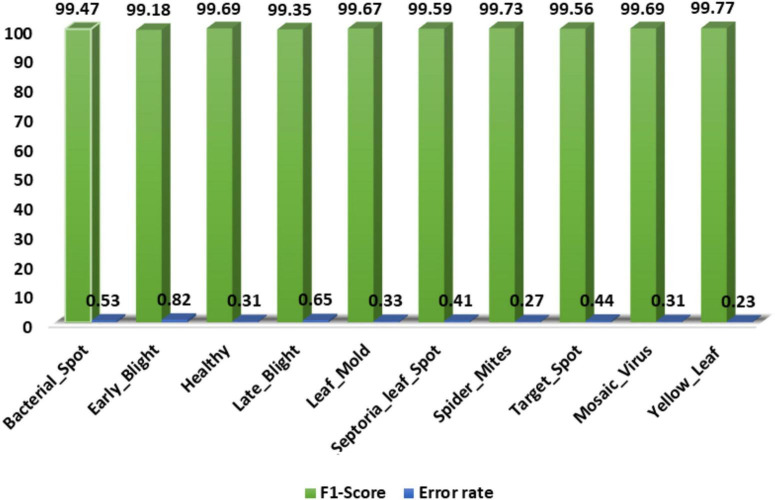
A pictorial depiction of the class-wise F1-score values obtained for the DenseNet-77-based CornerNet model.

Secondly, we show the calculated F1-score together with the error rate over the employed dataset and acquired values in Figure 10. The custom CornerNet model attains the average F1-score of 99.57% with the maximum and minimum error rates of 0.23 and 0.82%, respectively. The reported values demonstrate the robustness of the custom CornerNet model in locating and classifying all classes of tomato leaf disease efficiently.

**FIGURE 11:**
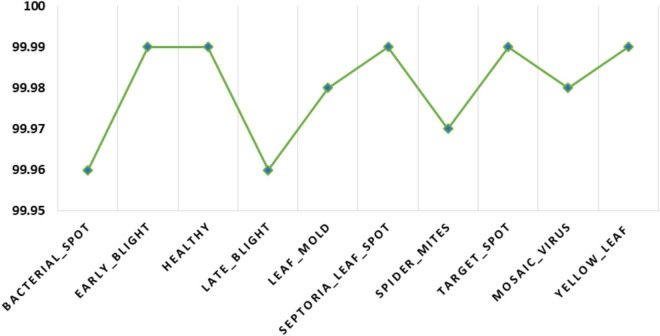
A pictorial depiction of the class-wise accuracy values obtained for the DenseNet-77-based CornerNet model.

Additionally, we have measured the class-wise accuracy values of the proposed technique and the acquired results are demonstrated in Figure 11. The introduced DenseNet-77-based CornerNet model attains the accuracy values of % for the 10 disease categories of the tomato plant and confirms the effectiveness of our approach.

**FIGURE 12:**
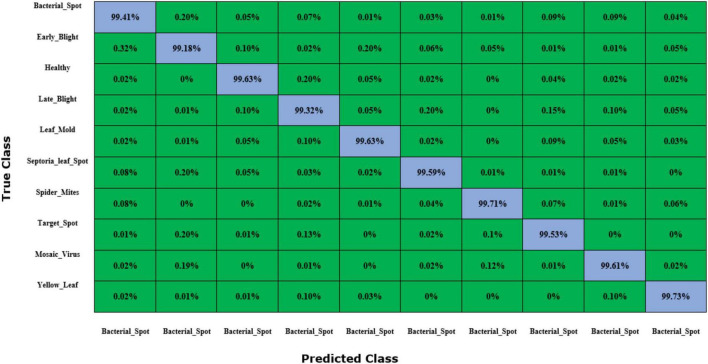
Confusion matrix results for tomato plant leaf diseases classification obtained using the DenseNet-77-based CornerNet model.

To further validate the class-wise accurateness of the introduced approach for distinguishing the numerous categories of plant leaf disease, we have created a confusion matrix (Figure 12). This plot can show the actual and estimated classes recognized by a model. The values shown in figure demonstrate that the custom CornerNet model is proficient at recognizing all classes of tomato plant leaf diseases due to its higher recall rate which empowered it to differentiate all categories reliably.

### Comparison with base approaches

In this section, we outline an experiment to compare the tomato plant leaf disease recognition capability of the improved CornerNet model against the base networks. We chose several well-known DL frameworks, i.e., GoogleNet, ResNet-101, Xception, VGG-19, and SE-ResNet50. The comparison is depicted in Table 3. The performance analysis shown in Table 3 illustrates that our technique is more accurate than the peer approaches. The DenseNet-77-based CornerNet model attains the highest results for the precision, recall, F1-score, and accuracy measures with the numeric count of 0.9962, 0.9953, 0.9957, and 99.98%, respectively. The second-highest results are reported by the SE-ResNet50 model with 0.9677, 0.9681, 0.9679, and 96.81% for the precision, recall, F1-score, and accuracy metrics, respectively. Moreover, the GoogleNet model attains the lowest results in classifying the leaf diseases of the tomato plant and attains the scores for precision, recall, F1-score, and accuracy measures of 0.8716, 0.8709, 0.8712, and 87.27%, respectively. The second-lowest values are attained by the Xception model with the numeric stats of 0.8825, 0.8814, 0.8819, and 88.16%. The comparison illustrates the effectiveness of our approach. Specifically, for the precision measurement, the selected methods have an average value of 0.9050, while the DenseNet-77-based CornerNet model acquires the value of 0.9962 and shows a performance gain of 9.12%. For the recall and F1-score, the selected models have attained the average numeric score of 0.9053 and 0.9091, while in comparative analysis the presented solution has shown the average recall and F1-score of 0.9953 and 0.9957, respectively. Therefore, we can demonstrate average performance gains for the recall and F1-score of 9 and 8.66%, respectively. Moreover, in terms of accuracy, the base models attain an average value of 90.56%. The proposed model attains 99.98% accuracy, representing a performance gain of 9.42%. Furthermore, we outline the time taken for each model. It should be noted that the proposed approach shows the minimum test time. The values show the efficacy of our work to better recognize the several classes of tomato plant leaf abnormalities. The basic cause of this better classification performance of the proposed improved CornerNet model is the employment of the DenseNet-77 model as the keypoints extractor. This uplifts the model to better select the image information to identify the affected areas of the plant leaves and better recognize the associated class.

**TABLE 3:** Comparison with other DL frameworks.

Model	Precision	Recall	F1-score	Accuracy (%)	Time (second)
GoogleNet	0.8716	0.8709	0.8712	87.27	0.65
ResNet-101	0.8995	0.9013	0.9004	90.13	1.21
Xception	0.8825	0.8814	0.8819	88.16	0.77
VGG-19	0.9039	0.9047	0.9243	90.42	1.56
SE-ResNet50	0.9677	0.9681	0.9679	96.81	0.57
Proposed	0.9962	0.9953	0.9957	99.98	0.22

### Performance evaluation with object detection approaches

We have employed an object detection-based model for the localization and classification of the tomato plant leaf diseases and compared the performance of the proposed approach with other object detection techniques. The major reason for performing this simulation was to verify the reliability of the proposed DenseNet-77-based CornerNet model against other competitor techniques while locating the diseased areas from the tomato plant leaves under the occurrence of noise, light alteration, color changes, size variations, etc.

To execute this analysis, we have chosen numerous well-known object detection approaches, namely the Fast-RCNN (Girshick, 2015), Faster-RCNN (Ren et al., 2016) YOLO (Redmon and Farhadi, 2018), the SSD (Liu et al., 2016), and CornerNet (Law and Deng, 2019) models. To measure the performance of the model, the mAP metric is used as it is the standard evaluation measure used by the researchers to assess the classification performance of the object detection techniques. Furthermore, we have compared the test time of models as well to evaluate the time complexities of the comparative approaches as well. The comparison shows the efficiency and effectiveness of our approach and is illustrated in Table 4. The results in Table 4 show that the proposed approach has the highest mAP score and lowest test time with a numeric score of 0.984 and 0.22 s, respectively. The second highest mAP score is the Faster-RCNN model with a numeric count of 0.884. However, it is computationally inefficient and shows a time complexity of 0.28 s due to its two-stage classification network architecture. The SSD model has the lowest mAP score of 0.883 and a test time of 0.27 s. Furthermore, this approach does not perform well for very small plant leaf sizes. The conventional CornerNet model also has less promising results with a mAP score of 0.883 and a test time of 0.25 s. Whereas, the DenseNet-77-based CornerNet approach better tackles the issues of existing object detection approaches for identifying and classifying the numerous categories of the tomato plant leaves and shows the highest results. The comparison object detection approaches have an average mAP value of 0.859, compared to 0.984 for the proposed algorithm. Therefore, we have attained an average performance gain of 12.42% for the mAP metric. The one-stage detection ability of the proposed approach reduces the network structure complexity which, in turn, gives it a computational advantage.

**TABLE 4:** Comparison with other object detection methods.

Models	mAP	Test time
Fast-RCNN	0.860	0.28
Faster-RCNN	0.884	0.28
YOLOv3	0.842	0.26
SSD	0.830	0.27
Hourglass-based-CornerNet	0.883	0.25
Proposed DenseNet-77-based CornerNet	0.984	0.22

### Model evaluation with the state-of-the-art methods

In this section, we have selected several new approaches (Tm et al., 2018; Kaur and Bhatia, 2019; Agarwal et al., 2020a) that worked for tomato plant leaf disease classification and have used analysis to compare the performance of the improved CornerNet model with them. For this purpose, we have utilized three standard measures: precision, recall, and accuracy. Agarwal et al. (2020a) proposed the EfficientNet model for the automated detection and classification of tomato plant leaf diseases and attained an average accuracy value of 91.20%. Tm et al. (2018) proposed a CNN framework for categorizing the affected area of plant leaves and demonstrated an accuracy value of 94%. Similarly, Kaur and Bhatia (2019) employed a deep learning framework for recognizing the 10 types of plant leaf diseases with an accuracy rate of 98.80%. Hence, the comparative analysis is depicted in Table 5 and illustrates that our work has attained the highest results for all selected performance measures. From Table 5, it can be viewed that the techniques in Tm et al. (2018), Kaur and Bhatia (2019), and Agarwal et al. (2020a) achieve the precision of 0.90, 0.9481, and 0.9880, respectively, whereas the introduced improved CornerNet model obtains the precision of 0.9962. This is the highest of all the reported numeric scores for the selected works. The improved CornerNet model gains the largest value of 0.9953 for the recall performance measure, while the approaches in Tm et al. (2018), Kaur and Bhatia (2019), and Agarwal et al. (2020a) have recall scores of 0.92, 0.9478, and 0.9880, respectively. Moreover, with regards to accuracy, the proposed approach gains the numeric score of 99.98% while the approaches in Tm et al. (2018), Kaur and Bhatia (2019), and Agarwal et al. (2020a) have accuracy values of 91.20, 94, and 98.80%, respectively. The peer works (Tm et al., 2018; Kaur and Bhatia, 2019; Agarwal et al., 2020a) have the average precision, recall, and accuracy values of 0.9453, 0.9519, and 94.67%, respectively, as opposed to 0.9962, 0.9953, and 99.97%, respectively, for the presented work. Therefore, the DenseNet-77-based CornerNet model provides performance gains of 5.08, 4.34, and 5.30% for the precision, recall, and accuracy evaluation measures.

**TABLE 5:** Comparison with the latest studies.

Approach	Precision	Recall	Accuracy (%)
Agarwal et al., 2020a	0.90	0.92	91.20
Tm et al., 2018	0.9481	0.9478	94
Kaur and Bhatia, 2019	0.9880	0.9880	98.80
Proposed	0.9962	0.9953	99.97

The reason for the competent classification results of the improved CornerNet model is that the techniques in Tm et al. (2018), Kaur and Bhatia (2019), and Agarwal et al. (2020a) are quite complex in network structure. This creates a framework over-fitting problem. The proposed solution is quite simple in structure and the employment of DenseNet-77 as the base network further empowered the CornerNet model to nominate a more reliable set of the sample feature vector. Such a model setting enhances its recognition ability by eliminating redundant information and reducing the model complexity. Further, the one-stage detection and classification ability of the CornerNet model prevents the framework from over-fitting issues and enables it to robustly deal with several image distortions like color, size, brightness, light variation, etc.

## Conclusion

The manual screening of tomato plant leaf diseases relies highly on domain experts to detect the detailed information from the samples under observation. AI-based solutions are trying to fill this gap by automating the manual screening system. However, excessive changes in the mass, color, and size of plant leaves, and the incidence of noise, blurring, and brightness variations in the images complicate the classification task. In this work, we have attempted to overcome the existing issues by proposing a deep learning-based approach namely the DenseNet-77-based CornerNet model. We have carried out extensive experimentations on a standard dataset, namely the PlantVillage, and have confirmed through both the visual and numeric computations that the proposed approach is both efficient and effective in recognizing tomato plant leaf disease. Furthermore, the proposed approach is capable of efficiently detecting the diseased area of the plant leaves from the distorted samples containing several image transformations. However, the approach shows small detection degradation for images with huge angular variations which will be a major focus of our future work. Moreover, we plan to test the proposed model on other plant diseases and evaluate other DL-based frameworks.

## Data availability statement

The original contributions presented in this study are included in the article/supplementary material, further inquiries can be directed to the corresponding author.

## Author contributions

SA: conceptualization, methodology, validation, software, supervision, and writing—reviewing and editing. MN: data curation, coding, validation, and writing—original draft preparation. Both authors contributed to the article and approved the submitted version.
